# Governance of prepaid consumption in China's sports industry based on collaborative governance theory: theoretical framework, realistic dilemmas, and practical pathways

**DOI:** 10.3389/fspor.2024.1506207

**Published:** 2025-01-09

**Authors:** Jianuo Su, Jinsheng He

**Affiliations:** Shanghai Normal University, Sport Department, Shanghai, China

**Keywords:** prepaid consumption, collaborative governance, sports industry, sport economy, sport business

## Abstract

The rapid development of prepaid consumption in China's sports industry has been hindered by fundamental issues such as operators’ blind expansion, lack of transparency in business information, absence of standard contract texts, and inadequate consumer rights protection. These challenges have led to business closures, unfair contract terms, difficulties in card refunds and transfers, and the excessive issuance of long-term or lifetime prepaid cards. These problems severely damage the industry's integrity and impede its healthy and sustainable development. Currently, the governance of prepaid consumption in the sports industry faces several realistic dilemmas, including unclear collaborative regulatory and law enforcement responsibilities of the government, an urgent need to improve the legal framework, ineffective government regulatory tools, difficulties in ensuring the safety of prepaid funds, and limitations in the credit evaluation system. By introducing the concept of collaborative governance, new ideas and practical solutions are proposed to address these issues. The practical pathways for collaborative governance of prepaid consumption in the sports industry based on this concept include constructing a collaborative regulatory mechanism to solidify the foundation of collaborative governance, improving legal regulations to broaden collaborative relief channels, innovating government regulatory tools to build a collaborative regulatory platform; optimizing fund custody mechanisms to ensure the safety of prepaid funds; and enhancing the credit regulatory system to refine the credit reward and punishment mechanism.

## 1 Introduction

In the rapidly changing landscape of consumer demands, consumption levels, and ideologies in China, prepaid consumption has emerged as a mutually beneficial strategy. However, in recent years, the high debt ratio has led to an increase in challenges such as “enterprise absconding,” “unfair terms and conditions,” obstacles to card refunds and transfers, and misuse of long-term to life-long prepaid cards (1). These issues not only violate consumer rights but also increase the government's regulatory responsibilities, significantly affecting market order and societal stability. Considering the extensive use of prepaid consumption in the sports industry, the large volume of cards issued, the wide consumer coverage, and the substantial scale of funds involved, any violation of consumer rights results in high and challenging costs for consumer rights protection. This situation is in stark contrast to the policy spirit of promoting the development of the sports industry as issued by relevant Chinese departments.

Despite substantial research on the legal regulation of prepaid consumption (2), risk control (3), and credit regulation (4), the sports industry presents unique challenges due to its longer consumption cycles, difficulty in quantification, and high subjectivity. Governance models suitable for general prepaid consumption often do not apply to the sports industry. Some scholars have investigated the legal regulation of prepaid fitness cards (5), contract disputes (6), and regulatory pathways (7). However, these studies are typically confined to the single payment method of fitness prepaid cards and fail to comprehensively address prepaid consumption issues in other areas of the sports industry. Consequently, more research is needed to provide a systematic and practical governance framework for prepaid consumption in the sports industry. Practically, various levels of government have actively explored governance in this area, but significant gaps still need to be established between actual outcomes and expected goals. This indicates that the current governance model needs to fully leverage the roles of all stakeholders, necessitating more effective governance strategies. To achieve effective governance of prepaid consumption in the sports industry, reliance on a single entity or governance method needs to be improved. Instead, a long-term governance mechanism should be established through collaborative governance.

Synergistic Governance Theory, an interdisciplinary approach combining Synergy Theory and Governance Theory, effectively engenders enthusiasm among government bodies, industry associations, operators, consumers, and other stakeholders. By fostering collaborative efforts in governance processes, it enhances the overall effectiveness of governance. The theory emphasizes the importance of coordinated cooperation among multiple parties in the management of public affairs, with the goal of maximizing governance efficiency. It has found wide application in various sports fields, including public sports services (8), sports events (9), and physical education (10). The core principle is to achieve governance goals through multi-party participation, resource sharing, and the distribution of power and responsibilities. Within this theoretical framework, this study aims to conduct a comprehensive analysis of the underlying causes of prepaid consumption in the sports industry and its governance challenges. Through literature reviews, logical reasoning, and other research methodologies, it further explores the construction of an efficient synergy model that involves the combined efforts of the government, industry associations, operators, and consumers. This approach aims to provide theoretical support for the sustainable development of prepaid consumption within the sports industry.

## 2 Theoretical framework for the collaborative governance of prepaid consumption in the sports industry

### 2.1 Connotation of collaborative governance theory

The connotation of collaborative governance theory is primarily embodied in the reform and development of governance subjects, governance objects, and their interrelations (11). At these three levels, collaborative governance showcases the characteristics of diversification, consultation, and flattening, providing a new theoretical perspective and practical path for enhancing governance effectiveness.

First, the diversification of governance subjects represents the most significant shift in collaborative governance compared to traditional governance models. In the traditional “single-center” governance theory, the government typically plays a leading role. As a regulator with limited rationality, its supervision often exhibits subjective tendencies. In contrast, collaborative governance theory emphasizes the participation of multiple subjects, particularly the integration of non-governmental and informal stakeholders. Respecting the autonomy of operators stimulates their enthusiasm for participation and improves the effectiveness of government regulatory policies (12). Operators can offer suggestions to the government based on real-world situations to ensure that policies strike a balance between protecting consumers' rights and respecting the autonomy of operators. This diversified composition of subjects disrupts the single, government-led governance model, emphasizing the cooperation of public sectors, enterprises, social organizations, and individuals. It aligns with the democratic ideal of citizen participation and provides new opportunities for the improvement and innovation of the governance system.

Secondly, the expansion of governance objects aligns with the trend of consultation in the governance of social public affairs. Traditional governance theory usually restricts the object of governance to matters directed by public policy. In contrast, collaborative governance underscores the universality of governance objects, encompassing all ways, methods, and processes based on consultation and consensus (13). This expansion helps to eliminate the antagonism and estrangement between the status, logic, and modes of governance subjects and objects, making the governance implementation process smoother and more efficient.

Thirdly, the flattening of subject relationships enhances the adaptability of collaborative governance in addressing complex issues. In collaborative governance, the relationship between governance subjects is no longer characterized by the traditional hierarchy of superiors and subordinates but rather by a partnership of equal cooperation. The diversification of authority in the governance process allows for either mandatory or voluntary collaboration, depending on the actual situation. This flexible and dynamic form of subject relationship enhances the adaptability of collaborative governance in complex environments and aids in efficiently addressing intricate social problems.

### 2.2 Analysis of the applicability of collaborative governance theory in the governance of prepaid consumption in the sports industry

In modern times, with the expansion of public administration, traditional administrative regulation has been unable to address increasingly complex economic and social problems. Particularly in the face of rapidly changing social needs and multiple interests, regulatory failures occur frequently. In response to this challenge, the government has gradually transformed into a service-oriented administration, seeking a more flexible and effective governance model. The model of industry autonomy has emerged, especially in fields such as network governance, food safety, and environmental protection (14). Industry self-management and self-supervision have gradually replaced traditional government regulation. However, in practice, this model exposes supervision vacuums and lacks effective mechanisms, leading to monopoly, interest transmission, and corruption (15). It highlights the limitations of the single-governance-subject model (16). Therefore, there is an urgent need for a pluralistic and comprehensive governance model to fill this gap. To address the issue of regulatory failure, the new public management movement proposed the theory of collaborative governance (17). This theory emphasizes the extensive participation of the government, industry associations, operators, and consumers, promoting information sharing and rule-making, thus forming a long-term and stable governance mechanism.

The construction of a multi-subject collaborative governance framework for prepaid consumption in the sports industry integrates the theories of complex adaptive systems (18), stakeholder theory (19), and network governance theory (20). Complex adaptive systems theory emphasizes the interdependence and dynamic influence of each component. Stakeholder theory highlights the importance of defining roles and responsibilities to foster cooperation among all parties. Network governance theory advocates for addressing the complexity and uncertainty of modern society through a multi-center and multi-level governance structure. Based on these theories, collaborative governance theory proposes abandoning the traditional top-down linear structure in favor of a grid-based, multi-subject co-governance network. This approach aims to balance multiple interests and create governance synergy, allowing all parties to enhance governance effectiveness through equal cooperation. Applying collaborative governance theory to prepaid consumption governance in the sports industry not only addresses the respective shortcomings of government and market mechanisms but also introduces a new approach to solving problems through multi-party coordination and cooperation. It balances the interests of all stakeholders, establishes a positive, cooperative relationship between officials and the public, and aligns with the governance concept of “co-construction, co-governance, and sharing” (21). This model transcends the limitations of traditional administrative orders, strengthens close cooperation among the government, operators, and consumers, and establishes a long-term, stable governance framework that ensures sustainability and effectiveness.

## 3 Root causes of prepaid consumption issues in the sports industry

Prepaid consumption in the sports industry refers to a consumption model where consumers pay a certain amount in advance to operators engaged in sports fitness, training, venues, and other services (including legal entities, non-legal organizations, or individual businesses). The operators then deduct the corresponding amount from the prepaid funds during consumption, typically using prepaid cards as vouchers (22). Consumers favor this consumption model for its flexibility and convenience, and it has become a new trading mode in the modern consumer market (23). However, due to its broad business scope, low market entry threshold, and high liquidity of funds, prepaid consumption faces several risks and challenges in its rapid development. The root causes of these issues in the sports industry are mainly reflected in four aspects:

First, the blind expansion of operators increases the risk of breaking the capital chain. Prepaid consumption is a financial business model where operators obtain funds through pre-sale cards to achieve zero-cost financing (24). Prepaid cards in the sports industry usually have a long term, resulting in a mismatch between the accumulation of funds and the provision of services. Operators often use this fund to expand, such as opening new stores. However, new stores cannot generate stable cash flow in the short term, exacerbating the time mismatch and deferral effect of funds. To maintain cash flow, operators continually attract new users, often neglecting service quality and internal management, leading to a decline in service attitude, product, and service standards (25). This results in decreased customer satisfaction, affecting consumers’ willingness to renew fees, and eventually forms a vicious cycle. For example, Kamo Yoga broke its capital chain in 2024 due to external factors like the epidemic and internal issues such as failing to adjust its market strategy in time, leading to the closure of all its stores. The company did not mention refund measures in its announcement, stating that it “cannot currently refund.”

Secondly, the opacity of business information makes it difficult for consumers to understand the actual business situation of operators. In recent years, due to the epidemic, rapid expansion, or poor management, some operators have broken their capital chains, and some even resort to false or exaggerated publicity to promote sales, inducing consumers to make blind purchases under conditions of asymmetric information. Consumer experiences that do not meet expectations quickly lead to disputes. Some operators even rush to sell cards before declaring bankruptcy, seriously disrupting market order and threatening consumer rights and interests (26). Although large listed companies are required to disclose their financial statements to provide some protection, it is often difficult for consumers to judge their actual financial situation. For example, taking the case of a trillion-dollar company, its turnover in 2019 was estimated to be between 1.9 billion and 2 billion yuan. The brand effect attracted a large amount of advance payments, which should have been a lifeline during the epidemic. However, the company is on the verge of bankruptcy due to the unknown whereabouts of the funds. Insiders suggest that prepaid funds may have been used for excessive store expansion, but there is a lack of apparent supervision and allocation of funds.

Thirdly, the absence of standardized contract texts and the dominant position of operators in format contracts lead to unequal rights and obligations between consumers and operators. Prepaid consumption is a contractual relationship; after the consumer makes an advance payment, the contract comes into effect, and the operator should perform the agreed service. However, in practice, many consumers complete transactions through payment and registration alone, needing a clear written contract (27). Model contracts in the sports industry have yet to be promoted nationwide. Many service contracts are oral agreements or need to clearly stipulate the quality of service, charging items, refund conditions, etc. It is difficult for consumers to understand the contract terms fully. Once disputes arise, operators often refuse to honor contracts with clauses such as “non-refundable balance” and “final right of interpretation,” leaving consumers' legitimate rights and interests unprotected.

Fourthly, there is a need for consumer rights relief organizations to ensure the effective implementation of consumer rights protection. The prepaid consumer market has the characteristics of public goods, and once established, it cannot exclude others from benefiting. Due to the “free rider” mentality, consumers lack the motivation to safeguard their rights actively, often expecting others to assume the responsibility of protecting their rights. At the same time, they seek low-cost benefits (28). This lack of self-protection awareness makes it more challenging to safeguard rights once mass incidents occur, further restricting the healthy development of the prepaid consumer market.

## 4 Practical dilemmas in governing prepaid consumption in the sports industry

### 4.1 Unclear responsibilities in government collaborative supervision and law enforcement

The supervision of sports prepaid consumption in China involves multiple departments, such as market supervision, sports administrative departments, and financial regulatory agencies. However, due to the unclear division of responsibilities among these departments, several issues arise in supervision, including overlapping responsibilities, blind spots in oversight, inefficiency, and a lack of effective division of labor and cooperation, making it difficult to form a cohesive regulatory force (29). The market supervision department is responsible for maintaining market order and protecting consumer rights but needs more specialized supervision methods and experience in the sports industry's subdivision field. The sports administrative department is responsible for industry standardization and development but needs more authority and means in the financial and legal supervision of prepaid consumption. Financial regulators focus on the security of prepaid funds but need to gain more knowledge of the specific operations of sports consumption. Additionally, a participation and co-governance mechanism involving all sectors of society has yet to be established (30). Due to their lack of independence, industry associations and performance guarantee institutions need help to exercise adequate self-discipline.

When issues arise in the prepaid consumption business of the sports industry, joint law enforcement by market supervision, commerce, public security, and sports administrative departments faces challenges such as unclear punishment powers and responsibilities, inadequate law enforcement, and difficulties in consumer rights protection (31). As a policy and competent department, the commercial department finds it challenging to implement measures effectively due to limited supervisory authority and resources and a lack of law enforcement power. Sports administrative departments and market supervision departments overlap in industry norms and market behavior supervision. In contrast, market supervision departments excel in handling consumer complaints but require the support of public security departments to combat illegal activities.

Due to the separation of supervision and law enforcement departments, the main body responsible for supervising the sports prepaid consumer market is unclear, the basis for punishment is ambiguous, and the discretionary space of law enforcement is difficult to control. This situation leaves the government with numerous “blank areas” in the supervision and governance of the prepaid consumer market. According to the Report on the Protection of Consumers’ Rights and Interests in the Field of Prepaid Consumption in 2023, many operators exploit regulatory loopholes and inadequate regulatory measures to issue and expand the issuance and use of prepaid consumer cards in violation of regulations.

### 4.2 Need for improvement in the legal framework

Currently, the legal regulation of prepaid consumption in the sports industry needs to be improved, resulting in a lack of regulatory basis and ambiguity in law enforcement. The existing law is overly general regarding qualification examination, operational supervision, subject responsibility, regulatory bodies, and dispute resolution, lacking specific operability and mandatory provisions (32). The Ministry of Commerce, responsible for the issuance and management of prepaid cards, has limited jurisdiction and needs to cover familiar entities such as individual businesses and non-enterprise legal persons (33). Article 53 of the Law of the People's Republic of China on the Protection of Consumer Rights and Interests only provides simple provisions on liability for breach of contract in prepaid consumption (34) without detailing performance or refund matters, leading to insufficient consumer rights protection (35, 36). In June 2024, the Supreme People's Court drafted the Interpretation on Several Questions Concerning the Application of Law in the Trial of Civil Disputes over Prepaid Consumption (Draft for Opinions), which clarified operators' responsibilities, including the return of advance payments and compensation for losses. However, it did not mandate that operators disclose contracts before consumers make payments, nor did it protect consumers' right to be informed and to choose.

Additionally, the dispute resolution mechanism remains to be clarified, with no dedicated mediation agency or expedited arbitration procedure and no clear standards for refunds or compensation when service quality could be better. In the absence of national laws and regulations, some local governments have started exploring solutions. For instance, Shanghai issued the Regulations on the Management of Single-purpose Prepaid Consumption Cards in 2018, the Beijing Sports Bureau and the Market Supervision and Administration Bureau issued a model text of prepaid service contracts in 2021, and the Beijing Sports Industry Prepaid Consumption Funds Supervision and Implementation Rules (Trial Implementation) were promulgated in 2023 to address gaps in local laws and regulations.

However, judicial remedies for prepaid consumption disputes still need to be clarified. Although the advance payment amounts are small, the process of safeguarding rights is complex, requiring consumers to provide their evidence and potentially hire lawyers, resulting in high litigation costs and time consumption (37). Besides litigation, consumers can seek assistance through consumer associations or administrative complaints, but these channels often need to be more efficient. Government departments such as the Consumer Protection Commission and the Market Supervision and Administration Bureau have limited supervisory means, needing more prior supervision and restraint measures, and their effectiveness in resolving issues could be better. Even if consumers win lawsuits, enforcement is challenging. Operators may need help to compensate or might maliciously transfer assets due to poor management, making it difficult to protect consumers' legitimate rights and interests.

### 4.3 Government regulatory tools not meeting expected effectiveness

In the process of collaborative supervision, numerous obstacles arise in information sharing, joint law enforcement, and case transfer due to the self-interest protection, geographical division, and technical limitations of various departments (38). As an important mechanism to address these issues, government regulatory platforms can foster cross-sectoral cooperation through the construction of functional platforms, enhance inter-departmental trust mechanisms, ensure data security and privacy protection, and thereby improve overall regulatory effectiveness. Currently, the Ministry of Commerce has established a nationwide single-purpose card business information system, utilizing information technology to supervise card-issuing enterprises, achieve real-time information submission, simplify the filing process, and ensure traceability of information content. Additionally, digital applications promote prepaid card filing, regulatory integration services, and security code adoption to strengthen source management, exemplified by Shanghai's single-purpose card collaborative supervision platform, Beijing's single-purpose prepaid card service system, and Taizhou's “Pay to Save Heart” initiative (39).

However, there are several areas for improvement in the actual operation of government supervision platforms. First, the speed and level of information development need to meet the needs of dynamic monitoring, data analysis, and intelligent early warning under the new circumstances, causing platform supervision to lag behind direct supervision. Monitoring platforms typically collect business information submitted by operators on a quarterly or annual basis, making real-time monitoring challenging. Second, the platform adopts a registration system reliant on voluntary declarations by operators, lacking procedures for qualification examination and content verification. This affects the effectiveness of information collection and often results in issues such as information distortion or excess operator funds. The absence of a mandatory registration system causes many operators to adopt a wait-and-see attitude or refuse to register to avoid supervision. This leads to a significant gap between the information docking coverage and the actual market situation. For instance, as of May 13, 2024, the completion rate of information docking for Hangzhou's single-purpose prepaid card digital supervision service platform was only 0.81%, indicating the platform's inefficiency. Third, the regulatory platform needs channels for multi-sectoral information interconnection. The liquidity and decentralization characteristics of prepaid consumption risks, combined with existing space-time management limitations, result in information lag. This issue necessitates exchange and interaction among the leading entities of the circulation chain. For example, the basic account, capital flow, and commercial credit information of prepaid consumer operators are typically controlled by financial institutions rather than competent authorities. As the “competent department of bank settlement accounts,” the People's Bank of China and its subordinate institutions find it challenging to supervise and disclose the flow of prepaid funds in real-time. Therefore, the government supervision platform needs to enhance information interconnection, particularly the transparency and sharing of business credit status.

### 4.4 Difficulty in ensuring the safety of prepaid funds

The fund depository mechanism is structured to inhibit operators from exploiting consumers' advance payments. This is achieved by mandating operators to deposit these payments into a bank's fund depository account. The Ministry of Commerce requires that the card-issuing operator signs a fund deposit agreement with the bank, thereby enabling the bank to oversee the deposit ratio and regularly report the funds' deposit status to the regulatory authority. Furthermore, banks are instructed to refuse any transfer of excessive funds (40). As it stands, China is progressively enhancing the qualification audit of prepaid consumption business entities, along with the mechanisms for auditing the number of issued cards and quotas. Cities like Beijing and Shanghai have proposed requirements for the deposit and management of prepaid funds and have established a mechanism for dynamic adjustment.

Nevertheless, the execution of the fund deposit and management mechanism faces specific issues: a majority of the market entities in the sports industry are small and medium-sized operators who often need more comprehensive fund management and financial transparency (41). These operators generally prefer to use the advance payment to expand their operations rather than subject their funds to government supervision. Despite being registered, some operators need to adhere to the requirements for depositing or using prepaid funds, thus making it challenging to comply with the stringent depository management prerequisites. The absence of a consistent regulatory mechanism within the industry results in varying implementation standards, thereby escalating the difficulty of executing the fund depository mechanism.

In cases where the operator suffers losses, declares bankruptcy, or engages in deliberate fraud, the depository funds or reserve funds can be utilized to compensate consumers. However, existing laws and regulations do not clearly define the party responsible for compensation and the procedures involved, thus necessitating an urgent resolution to the issues concerning compensation procedures and responsibilities following an operator's business failure. Moreover, the fund deposit and management mechanism lacks a capital risk early warning system. The sports industry, in general, needs an effective capital risk early warning mechanism for reasons including inadequate regulatory systems and technical methods to monitor real-time fund flow and usage and the inability to detect and warn against potential capital risks promptly. Professional risk management personnel and technical support are necessary for enterprises to establish and maintain an effective risk early warning system.

### 4.5 Limitations of the credit evaluation system

The Market Supervision and Administration Bureau, along with other departments, has yet to establish a comprehensive credit evaluation and supervision mechanism for prepaid consumer operators. This deficiency results in an inability to accurately assess the creditworthiness of these operators, thereby preventing the implementation of appropriate restraint measures based on credit ratings. The modern economy is fundamentally a credit economy, with credit playing a pivotal role in the allocation of market resources and forming the crux of economic and social operations (42). An effective credit evaluation system is necessary for consumers to gain insight into the actual operational conditions and service quality of operators when procuring prepaid consumer services. Consumers are often left to indirectly assess their credit level through superficial factors such as location, decoration, and equipment, making informed decisions difficult (43). The lack of a credit evaluation system means the credit rating of operators is mainly contingent on personal ethics. If short-term benefits sway operators and operate in bad faith—for instance, through the use of unreasonable terms in the card handling process, unfulfilled service commitments, or significant discrepancies in service quality—it becomes challenging for regulatory authorities to acquire comprehensive credit information. This limitation can lead to non-targeted supervision, ineffective punishment for dishonest operators, and delays in rectifying and addressing violations.

Currently, the credit evaluation system needs several issues: the scope of responsibility within the credit restraint mechanism is narrow and confined only to the legal representative of the operator. Frequent evasions of credit punishment through the use of other identities or changes in legal representation are common occurrences. Credit information is often scattered or missing, and the issuance of prepaid cards does not require registration. The credit status of operators needs to be more effectively linked with the entry or exit mechanism of the prepaid card market, leading to a need for more regulatory information during cancellations or changes. This lack of information makes effective credit punishment for operators, legal representatives, and actual controllers challenging. There is also inadequate punishment for dishonesty, with a need for more innovation in punishment techniques and methods, which are mainly fines. Such measures need to be revised for the rapidly evolving business models and technological advancements, and the low cost of dishonesty fails to deter malpractices by operators. Additionally, an incentive mechanism encouraging good faith has yet to be established, resulting in operators lacking the motivation for self-regulation and fostering a market environment that values trust.

## 5 Practical pathways for the collaborative governance of prepaid consumption in the sports industry

### 5.1 Construct a collaborative supervision mechanism to solidify the foundation of collaborative governance

By applying the theory of collaborative governance, we can reshape the cooperative relationship among government, industry, and market in regulation and avoid the excessive dependence of the government on “institutional ignorance.” The government should value the opinions of trade associations, operators, and consumers, making decisions that align with market demand through information exchange and consultation (44). Industry associations, consumer organizations, and performance guarantee agencies can leverage their professional advantages to reduce regulatory costs, accumulate risk knowledge, and enable the government to focus on macro-planning and coordination. Through the co-governance model combining business entities' self-discipline and government-guaranteed responsibility, the self-inspection and self-correction obligations of prepaid consumer operators are clarified. This approach encourages their sense of social responsibility, corrects cognitive biases regarding risks, establishes responsibility ethics and behavioral norms, and purifies the industry's ecological environment.

#### 5.1.1 Clarify the subjects of collaborative supervision and their specific responsibilities

**Figure 1:**
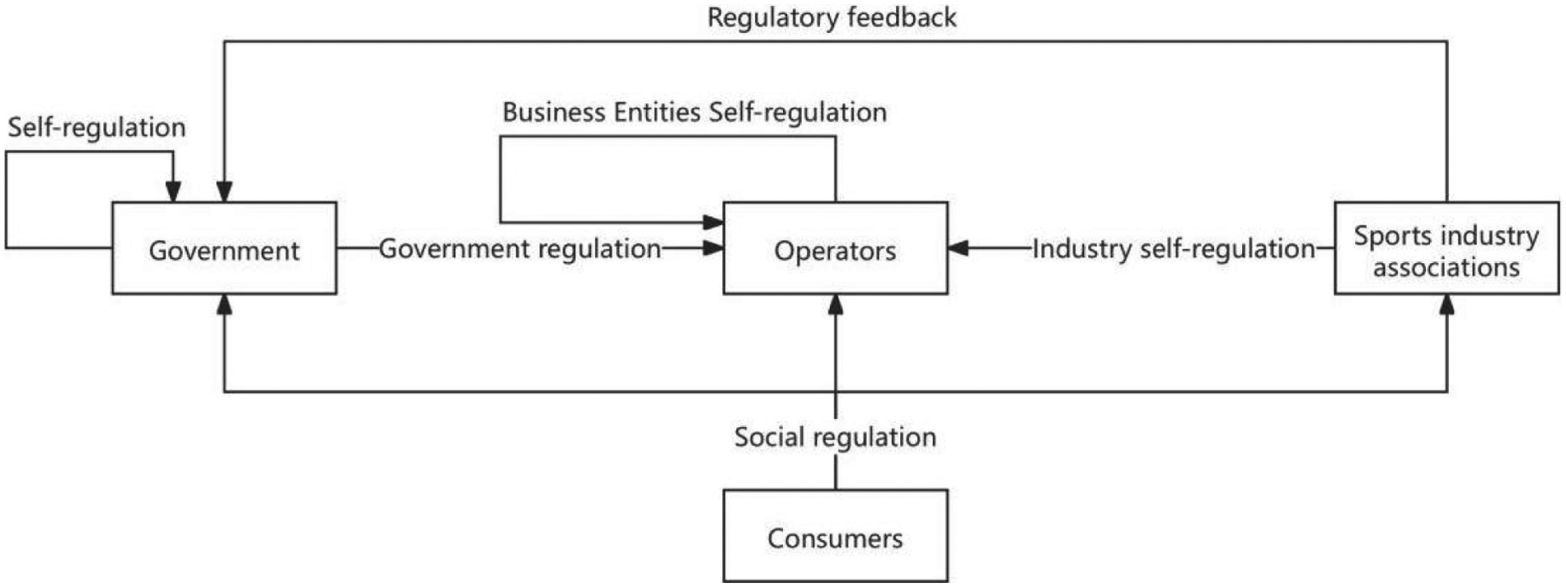
Relationship diagram of multiple entities in the collaborative governance model (45).

In the sports industry, collaborative governance enhances the synergy, pertinence, and fairness of prepaid consumption supervision through equal participation, mutual trust, and cooperation of multiple entities. The government, operators, sports industry associations, and consumers bear different yet complementary responsibilities: the government maximizes the public interest through regulation and control with legitimacy and coercive force; operators, as the market's main body, directly influence market operations and consumer rights, and balancing self-discipline with supervision helps enhance market competitiveness and integrity; sports industry associations, by guiding and standardizing industry behavior, can compensate for the government's micro-level supervisory shortcomings and improve the industry's overall level and self-discipline ability. Consumers, as the ultimate embodiment of market demand, promote the healthy development of operators and the industry through positive feedback and supervision. The network of relationships among multiple entities in the collaborative governance model of prepaid consumption in the sports industry is illustrated in Figure 1 below.

Government supervision departments should fully utilize their public power advantages to fulfill their responsibilities in formulating laws and regulations, macro-coordination, and policy implementation in the prepaid consumer market of the sports industry. They should strengthen supervision, increase the number of supervisors, and improve the level of oversight. It is essential to streamline the relationship between supervision and law enforcement, achieve closed-loop management, avoid the limitation of functional departments “only in charge of issuance and filing,” and clarify the regulatory boundaries of new formats and new consumption modes. In accordance with the single-purpose prepaid card regulations of local governments, specific implementation rules should be formulated to clarify the issuance qualifications of prepaid cards, fund deposits, and credit ratings, thereby providing industry norms (46). Coordination among sports industry associations, operators, and consumers should be facilitated through regular meetings to analyze and discuss disputes, successful cases, and potential risks in the industry, thereby enhancing the synergy of multi-governance (47).

Operators should adhere to laws, regulations, and industry self-discipline norms, establish a culture of honest management, assume social responsibilities, improve service quality through innovative business models, strengthen self-regulation, enhance internal management systems, and improve financial, member management, and complaint handling mechanisms. They must eliminate false advertising, malicious marketing, and information leakage and disclose business information in a timely manner to protect consumers' right to know.

As a self-discipline organization, sports industry associations should promote the construction of industry self-discipline mechanisms, standardize industry development, and enhance the overall level of self-discipline. They should formulate industry self-discipline conventions based on the implementation rules of the prepaid card market, propose specific requirements for the sale, use, and refund of prepaid cards, and enhance industry norms. Regular inspections of member operators should be conducted, violations should be penalized, and regulatory authorities should be notified to reduce the pressure of government supervision. Associations should organize operators to formulate and adhere to self-discipline norms, provide industry information, business consulting, and training services, and improve the level of operation and risk resistance. They should lead the establishment of peer mutual insurance bodies, allowing enterprises in the same industry that meet the standards to voluntarily adopt mutual guarantee forms and sign mutual insurance contracts with established industry organizations.

Consumers are not only the protected but also the regulators in the prepaid consumption of the sports industry. They should establish legal awareness, rational consumption, and rights protection awareness, understand the status of operators and the risks of prepaid cards, make prudent consumption decisions, and safeguard their rights and interests. When illegal acts are discovered, they should report them to regulatory authorities, sports industry associations, or consumer associations, or warn other consumers through consumer evaluations to urge operators to correct them promptly and promote healthy competition and market development.

#### 5.1.2 Establish an effective accountability mechanism to prevent the abuse of regulatory power

Although collaborative governance has significant advantages in balancing the interests of multiple subjects and mobilizing operators' enthusiasm to participate in governance, it still has limitations in coordination and implementation. To address these deficiencies, it is necessary to build a composite regulatory responsibility system covering multiple regulatory subjects. This system should ensure that social organizations and individuals bear corresponding responsibilities according to the law when they abuse social power and that state organs or authorized organizations exercise their power legally (48). Establishing a regulatory responsibility system helps enhance the consciousness of responsibility in multiple regulatory subjects and prevents the phenomenon of regulatory slackness caused by power without responsibility.

Government supervision departments should regularly accept supervision from their superiors, internal departments, and society. They should be held accountable and impose administrative sanctions on public servants who fail to perform their duties, neglect their duties, or excessively interfere with the autonomy of market participants. Sports industry associations should improve their internal supervision structures, establish accountability mechanisms, and accept government and societal supervision through regular information disclosures. In case of any violation of laws or regulations, regulatory authorities may take measures such as administrative interviews or rectification within a time limit to urge corrections. For operators who fail to actively perform their self-regulation duties, resulting in violations of laws and regulations, government regulatory authorities may restrict their activities through administrative penalties and measures such as lowering their credit rating, requiring them to rectify within a time limit. Sports industry associations can be held accountable through industry notification and criticism. Since consumers are mainly protected within multiple regulatory subjects, it is challenging to investigate their regulatory responsibilities. However, consumers can be encouraged to actively perform their regulatory responsibilities through publicity and education, simplifying the complaint reporting process and increasing the weight of consumer evaluations in the credit rating system.

### 5.2 Improve legal regulations and broaden collaborative relief channels

The key to governing prepaid consumption lies in constructing legal regulations that align with market development. Governments at all levels should expedite the formulation of local regulations or specific regulatory measures in accordance with the Regulations on the Implementation of the Consumer Rights and Interests Protection Law. They should establish management systems that include access, filing, deposit, and management mechanisms for prepaid funds and clarify business qualifications, regulatory departments, and legal responsibilities (49). The legal status of contracts and the rights and obligations of both parties should be established, and the promotional actions of sales personnel should be regulated to provide legal protection for consumers. Sports industry associations should promote model contract texts, eliminate the adverse effects of unfair clauses on consumers, establish a robust complaint and reporting mechanism, and ensure timely detection and resolution of issues during contract performance. Additionally, the legislative process should clarify the division and connection of responsibilities among industry authorities, administrative law enforcement departments, and comprehensive supervision departments. It is recommended that local government leaders establish a special task force to promote departmental cooperation.

To protect consumers from dishonest operators, existing regulations and relief channels must be optimized. Under a collaborative governance model, consumers and operators enjoy corresponding rights and interests equally, avoiding favoritism. To prevent unfair treatment and infringement, diversified regulation and relief methods should be established to ensure the adequate protection of both parties' legitimate rights and interests (50).

At the national level, state power organs should play a crucial safeguarding role. First, in terms of administrative relief, smooth channels for complaints, reports, and reconsideration should be ensured. A dedicated service hotline or platform should be set up to handle prepaid consumer disputes in the sports industry, and administrative relief procedures should be optimized through online processing to improve efficiency. Government regulatory authorities may also lead in establishing a dispute mediation committee composed of sports industry associations, operator representatives, and consumers to handle industry disputes and provide professional relief. Second, in terms of judicial relief, the People's Court should develop an efficient handling mechanism tailored to the characteristics of prepaid consumption disputes, establish a unique dispute resolution group, promote pre-litigation mediation, and maximize the legitimate demands of the parties. Concurrently, the court should establish an online litigation platform to offer convenient online acceptance, trial, and adjudication services.

At the societal level, priority should be given to resolving disputes through self-coordination and internal resolution. First, as self-regulatory organizations, sports industry associations should actively mediate prepaid consumption disputes in the sports industry, providing fair, professional, and efficient mediation services to resolve disputes within the industry. Second, operators should establish internal dispute mediation mechanisms to address consumer rights and interests disputes promptly, safeguarding consumers' rights and interests as well as corporate reputation, and set up dedicated consumer complaint channels to improve operators' credit ratings. Third, consumer associations should actively offer complaint acceptance and rights protection advisory services to help consumers safeguard their legitimate rights and interests.

### 5.3 Innovate government regulatory tools and build a collaborative supervision platform

The collaborative governance of multiple stakeholders relies on the seamless flow of information and cooperation. It is essential to establish a governmental supervision platform for information sharing, communication, and collaboration. This platform should strengthen information exchange among sports administrative departments, sports industry associations, operators, and consumers, thereby avoiding inefficient cooperation caused by mistrust (51). The regulatory platform must enhance the procedural rules of “multi-coordination,” promote a culture of comprehensive staff and social co-governance, clarify the roles of market and industry entities within the platform, and grant them rights to participate in policy formulation and risk decision-making. For instance, performance insurance institutions should be granted greater rights to information and decision-making powers.

Additionally, sports industry associations should be empowered to supervise and rate the information disclosure of prepaid consumer operators, thereby reducing the regulatory burden on authorities. Establish rules for rational consultation and multiple exchanges to ensure that social stakeholders participate equally in policy demonstration and supervision. In the process of enhancing the regulatory platform's functionality, it is necessary to refine the legal regulations governing its framework. This includes instituting mandatory filing obligations for operators through legislation, incorporating operators above a specific scale into the standard filing system, and improving the platform's oversight and efficiency. Institutional norms should be established to delineate the specific functions and responsibilities of “department coordination,” thereby enhancing the platform's credit risk warning interconnection capability.

To optimize information collection, publicity, and data processing on the supervision platform, we should advocate for the application of blockchain technology across all aspects of the platform (52). The regulatory platform can employ a unified blockchain system to record and monitor business transactions, capital flows, social credit, litigation, and other information related to prepaid consumer operators. This information should be accessible through the platform's publicity channels to stakeholders such as contracting consumers, performance guarantee agencies, and regulatory authorities (53). Furthermore, the regulatory platform should introduce a performance dispute resolution module. This module should include a unified and standardized dispute resolution mechanism to preemptively address prepaid consumer disputes, streamline the dispute resolution process, and promote the effective handling of complaints. The traceability of the platform's information content should encourage all parties involved in disputes to provide authoritative, authentic, and relevant evidence, standardizing the content and procedures of evidence collection and proof. The dispute resolution module should also oversee the entire settlement process, integrating the Internet court with the platform to facilitate the entire cycle of mediation, evidence collection, and litigation for prepaid consumer performance disputes (54).

### 5.4 Optimize the fund custody mechanism to ensure the safety of prepaid funds

An innovative fund supervision model requires the establishment of a mandatory fund deposit mechanism for all prepaid consumer operators, ensuring compensation for consumers through bank deposit funds in case of disputes. Under the guidance of the sports administrative department, sports industry operators must sign a service agreement with a depository bank detailing the depository mode, appropriation rules, working time limits, and responsibilities of all parties involved. The depository bank is prohibited from embezzling or misappropriating the prepaid funds and from collecting any depository fees from operators and consumers. The financial supervision department must regularly share information regarding the deposit and management of prepaid funds and associated risks with the sports administrative department. To enhance fund security, a unified fund monitoring system should be established, along with a government-enterprise linkage early warning mechanism involving government departments, payment institutions, and commercial platforms. This system would restrict fund transfers and withdrawals from various channels and strengthen consumer alerts at the payment and consumption stages. Real-time interaction and publicity between government supervision platforms (tracking anomalies like social security suspensions, complaints, relocations, etc.) and payment platforms such as UnionPay, Alipay, WeChat, and third-party commercial platforms like Taobao and Meituan Dianping should be explored to mitigate risks through a linkage warning system.

A government-led market-oriented solution involves using the government supervision platform as a third-party depository institution to monitor and provide early warnings of capital flow through an extensive data system for prepaid consumption supervision, thus creating a safer consumption model. This approach may include “enjoy now, pay later” options, allowing consumers to receive services before making periodic payments, thereby reducing financial pressure and mitigating market risk through third-party guarantees to channel funds appropriately. To address market changes and risk management needs, a dynamic adjustment mechanism for the proportion of deposits and management of advance funds should be established, with different proportions set for different operators. The “Collaborative Supervision Service Platform” in Shanghai, which integrates operators, consumers, banks, and regulators, can serve as a reference despite its limitations in capital supervision scope and advance payment proportion. Additionally, the “Chaoyang Pre-deposit Treasure” WeChat program in Beijing can be considered, offering an interface for operators and consumers where the bank thoroughly monitors advance payments. The operator fulfills their obligations, the consumer acknowledges completion, and the bank transfers the cost to the operator's account for a one-time settlement. The experiences of Shanghai and Beijing provide valuable insights for improving China's prepaid fund supervision system and should be utilized as references.

### 5.5 Enhance the credit supervision system and refine the credit reward and punishment mechanism

The key to effective credit supervision lies in the incentive and punishment system for breaches of faith, which directly affects the rights and obligations of market participants (55). Trustworthiness incentives provide benefits to market participants with good credit, including reputation, opportunities, conditions, and procedural incentives. Punishments for dishonesty impose adverse consequences on those who break their promises to achieve the purpose of punishment and warning. The implementation of differentiated regulatory strategies should be based on specific circumstances and ensure that the means and measures are fully justified. There should be reasonable grounds for differential treatment of operators. According to the “pyramid of enforcement,” the executive should begin with recommendations and persuasive measures and escalate to severe sanctions (35, 36). However, current punishments for dishonesty tend to be large-scale and automated. Once included in the list of dishonest entities, a series of disciplinary measures are automatically triggered, deviating from the pyramid model and ignoring precise law enforcement. In the future, punishments for dishonesty should be more refined, with measures matched according to the specific circumstances of dishonesty to achieve “excessive punishment.” From the perspective of cost and benefit, punishments for dishonesty should balance the marginal cost and marginal benefit, determine the optimal scale of punishment, and ensure the effective deterrence of measures (56). Given that the legal effect of credit rewards and punishments still needs to be clarified, it is necessary to carefully evaluate the impact of various measures during their formulation and implementation. It is also important to be vigilant about repeated and malicious complaints caused by human operation, as well as the damage to the interests of operators caused by excessive rights protection and false evaluation. A fair and just consumer credit evaluation system is crucial for promoting the protection of consumer rights and interests. Therefore, it is suggested that consumers present their original identity card when safeguarding their rights. Such behavior should be entered into the consumer credit evaluation system to facilitate the supervision of consumer rights protection behavior.

We should leverage the advantages of collaborative regulation, promoting the participation of sports industry associations, third-party credit rating agencies, operators, and consumers under the coordination of sports administrative departments (57). A scientific and reasonable credit evaluation system for sports industry operators should be formulated. Through dynamic evaluation and publication of credit rating results, we should strengthen the restraint on operators, improve information transparency, and help consumers make rational choices. Sports administrative departments and industry associations should establish a positive credit incentive mechanism, commend and reward honest operators, simplify the process of handling affairs, enhance market publicity, foster healthy competition, and improve the credit level of the industry. Through the government supervision platform, the credit information of the national sports industry can be shared, dishonest entities exposed and blocked, their prepaid consumption and operation qualifications canceled, their participation in government procurement restricted, and their credit status linked to the application conditions of loans and insurance, thereby achieving joint punishment (58). Strengthen the linkage of industry credit collection, organize special rectification and joint law enforcement, build an early risk warning mechanism for business entities, monitor key dishonest operators, and prevent significant risks.

## 6 Conclusion

As public demand for health and sports diversifies, prepaid consumption in China's sports industry is experiencing rapid growth. However, this has led to prominent issues such as contract disputes, consumer fraud, and capital security. To enhance the governance efficiency of prepaid consumption, optimize the market environment, and foster the industry's sustainable development, this study elucidates the essence of collaborative governance theory. It delves into the root causes and practical challenges of governing prepaid consumption in the sports industry. The study proposes a practical pathway of collaborative governance that includes strengthening the governance foundation via a collaborative supervision mechanism, advancing the rule of law via government legislation, augmenting supervision efficiency via a collaborative supervision platform, guaranteeing the safety of consumers' prepaid funds via a fund deposit mechanism, and constraining commercial behavior via credit supervision. The aim is to construct a collaborative governance system for prepaid consumption in the sports industry, which encompasses government department leadership, market industry self-discipline, business entity integrity, and social consumer participation.

This study offers a novel theoretical perspective and presents a specific pathway for all stakeholders to enhance governance through collaboration, thus bridging the gap in systematic research on the governance of prepaid consumption in the sports industry both domestically and internationally. However, this study, while proposing a theoretical governance pathway, acknowledges the absence of empirical research support. Future studies could further validate the feasibility and effectiveness of the suggested governance pathway using case studies and empirical data. This study will likely provide theoretical backing for the sustainable development of prepaid consumption in the sports industry and furnish a foundation for the creation and refinement of relevant policies.
